# P354: Beninese networks to improve patient safety and the right to health (REBASEP): objectives- strategies- activities- results- challengies and perspectives

**DOI:** 10.1186/2047-2994-2-S1-P354

**Published:** 2013-06-20

**Authors:** A Taibatou Biao

**Affiliations:** 1 Secretary REBASEP, Cotonou, Benin

## Introduction

The African countries have experienced a long delay in the implementation of the recommendations of the World Alliance for Patient Safety. Much effort has been deployed in this direction by civil society organizations. It is within this framework that had been established in June 2009 under the sponsorship of RIPAQS (International Network for the Improvement of Planning and Quality of Care) the REBASEP.

## Objectives

Working for patient safety; create a permanent framework for dialogue and exchange, work to minimize the adverse effects; promote quality care to meet one of the fundamental rights of the human being who has the right to health.

Strategies: Establish a national executive consists of patients who underwent side effects and parents of patients developing a roadmap for 3 years, other CSOs call for the same objectives as the REBASEP , organization of conferences, discussion meetings and other exchanges.

## Methods

Organization of advocacy and awareness, participation in sessions meeting at the municipal level, involvement in a series of training at the House of civil society, drafting, publishing network in a local newspaper, survey in hospitals to identify and listen to some victims, several approaches for recognition with the former Minister of Health and as the Director of HZ Sakété.

## Results

Health care workers are aware of the issue of patient safety that preoccupies many patients.

## Challenges

REBASEP has difficulty operating, not enough institutional support, no funding available to support its projects.

## Conclusion

Search for donors to support its projects seeking technical and financial partners, building partnerships with the Ministry of Health.

The **REBASEP** wants and can play an important role in improving patient safety in Benin, it suffices that the capacity of its members to be strengthened.

## Disclosure of interest

None declared.

